# Establishment of minimally invasive ventral hernia repair with extraperitoneal mesh placement in a primary care hospital using the robotic platform

**DOI:** 10.3389/fsurg.2022.964643

**Published:** 2022-11-23

**Authors:** Katrin Bauer, Frank Heinzelmann, Robert Vogel, Peter Büchler, Björn Mück

**Affiliations:** Klinikum Kempten - Klinikverbund Allgäu, Kempten, Germany

**Keywords:** ventral hernia repair, robotic hernia repair, retromuscular mesh placement, preperitoneal mesh placement, minimally invasive surgery

## Abstract

**Background:**

The progressive availability of robotic surgical systems opens new perspectives in abdominal wall surgery due to excellent visibility and dexterity of instruments. While complex hernias until today were treated primarily through an open access, we evaluated if this promising technology is suitable for treating the entire spectrum of a hernia center, including complex hernias.

**Material/methods:**

In 2017, minimally invasive hernia surgery with extraperitoneal mesh placement was started in Kempten hospital. Since 2019, a Da Vinci X system has been available for this purpose. In order to observe the process of transition we retrospectively analyzed all patients who underwent ventral hernia repair in the department of general and visceral surgery at our hospital between January 2016 and December 2020 and were indicated for mesh implantation.

**Results:**

In 2016, the percentage of minimally invasive procedures was 37.3%. In all of these cases an intraperitoneal mesh was implanted into the abdominal cavity. Open surgery was performed in 62.7%, of which an a retromuscular mesh was implanted in 75.7%, an intraperitoneal mesh in 21.6%, and an onlay mesh in 2.7%. In 2020, minimally invasive surgery accounted for 87.5%, of which 85.7% were performed robotically and 14.3 laparoscopically. In 94.3% of these minimally invasively treated patients the mesh was implanted in extraperitoneal position (75.8% in retromuscular and 24.2% in preperitoneal position). The percentage of complex hernias increased from 20.3% to 35.0% during the same period.

**Conclusion:**

The majority of ventral hernia procedures can be performed safely using the robot in a minimally invasive technique with extraperitoneal mesh placement without leading to an increase in complications. Robotically-assisted hernia repair is a promising new technique that is also practical for complex hernias.

## Introduction

Minimally invasive surgery (MIS) has been established in various fields of visceral surgery within last two decades. This trend becomes apparent also in Germany. The availability of robotic systems has increased this development in most areas of abdominal surgery.

In hernia surgery, laparoscopic surgery has become widespread since the initial description of laparoscopic intra peritoneal onlay mesh (IPOM) placement in 1993 by Karl LeBlanc (1). The intraperitoneal mesh position of this procedure differs from that of the retromuscular “sublay” procedure, which usually is still performed using open surgery. Meanwhile systematic reviews showed that retromuscular mesh placement is the ideal mesh position and shows the best results in terms of recurrence and complications (2, 3).

In Germany, these findings have in turn led to a decrease in the proportion of patients undergoing minimally invasive surgery in favor of open surgery over the past 10 years. Köckerling et al. reported a significant decrease in the laparoscopic IPOM procedure from 33.8% to 21.0% between 2013 and 2019 analyzing the Herniamed data of incisional hernia whilst the sublay procedure with open access has increased from 32.1% in 2013 to 41.4% in 2019 (4).

Recently several extra- and transperitoneal minimally invasive techniques with retromuscular mesh placement have been described (5–9). However, since these procedures are technically very demanding, they are not yet widely used. In 2019 in Germany this group of procedures did not yet play a significant role. In the analysis of Köckerling the minimally invasively performed procedures using retromuscular/extraperitoneal mesh placement were subsumed with other techniques in the group “others” which amounted to only 10% (4).

In recent years, several robotic adaptations of these techniques have been described (10, 11). While retrospective studies about robotically assisted procedures in areas where there was already a high proportion of MIS, such as colorectal resections, show similar advantages as known of conventional laparoscopic surgery (12), robotics in hernia repair should rather be compared to open surgery, since laparo-endoscopic surgery with extraperitoneal mesh placement is practically irrelevant in clinical practice. Since the advantages of the minimally invasive approach are well known this comparison could result in a greater difference in benefit to patients in the field of hernia surgery.

We’ve established the robotically-assisted technique in our hospital in 2019. Ever since it has been in use for treatment of abdominal wall hernias. Within the framework of a feasibility study we already analyzed retrospectively the first 50 cases of robotically-assisted ventral hernia surgery (13). The aim of this work is to examine whether the increase in proportion of minimally invasive hernias with extraperitoneal mesh placement within the spectrum of a hernia center is due to the use of the robot and in particular, to analyze whether the robot is also suitable in treatment of complex ventral hernias.

## Methods

Since September 2015, given an informed consent, the data of all operated patients have been meticulously recorded into the Herniamed database (14) for quality assurance purposes. The study included all inpatients who underwent surgery for a ventral hernia with indication for mesh implantation in the period January 2016 until December 2020 at Kempten hospital. The term ventral hernia includes incisional hernias as well as primary hernias of the abdominal wall, such as umbilical hernias, epigastric hernias and spieghelian hernias. The data of all patients were analyzed using the hospital information system and the Herniamed database (14). The following data were collected retrospectively: perioperative parameters (surgical procedure, complexity of the procedure, intraoperative complications), postoperative parameters (type of complication, complication rate, reoperation rate within the first 6 weeks after surgery) and hernia-specific parameters (hernia type, hernia size, hernia location, mesh position).

Hernia findings were classified in analogy to the classification of the European Hernia Society (EHS) (15). Mesh positions were classified based on the classification of abdominal wall planes by Parker (16). The retrorectus and retromuscular mesh positions were combined in the retromuscular group. The complexity of the procedures was categorized based on the criteria of the publication by Slater et al. (17). Perioperative morbidity was graded according to Clavien–Dindo classification (18). In 2017, minimally invasive ventral hernia repair with extraperitoneal mesh implantation was started in Kempten hospital. The laparoendoscopic techniques used were eTEP (enhanced view total extraperitoneal plasty) and eMILOS (endoscopic Mini/Less-open-Sublay) as retrorectal, or retromuscular procedures in combination with a Transversus Abdominis Release (TAR) and ventral TAPP (Trans Abdominal Pre Peritoneal) as preperitoneal procedure. Since 2019, a Da Vinci X system (Intuitive Surgical, Sunnyvale CA, United States) has been available for this procedure. Because the participating surgeons had no prior robotic experience, robotic surgery was initially limited to smaller hernias of size EHS W1 to EHS W2, with both lateral and medial findings. The surgical procedures used were the retromuscular TARUP (Transabdominal Retromuscular Umbilical Prosthetic hernia repair) technique and the ventral TAPP approach as the preperitoneal procedure. After 20 procedures, larger findings requiring TAR and techniques with extraperitoneal access (eTEP) were also operated on. Robotic training was supported by Intuitive Surgical. Several surgical courses were attended at training centers where the procedures could be practiced first on cadavers. A proctor from Intuitive Surgical was present during the first ventral hernia operation and the first TAR.

## Results

**Figure 1:**
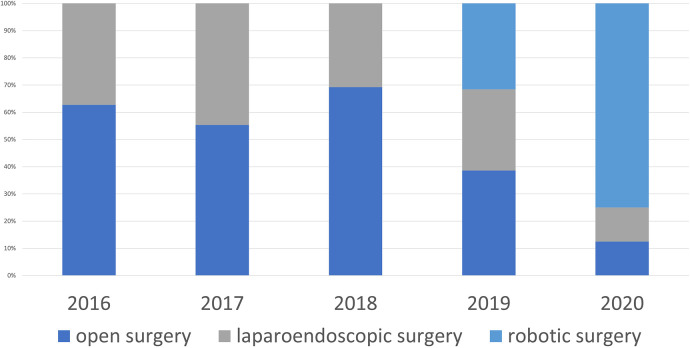
Method of operation.

**Figure 2:**
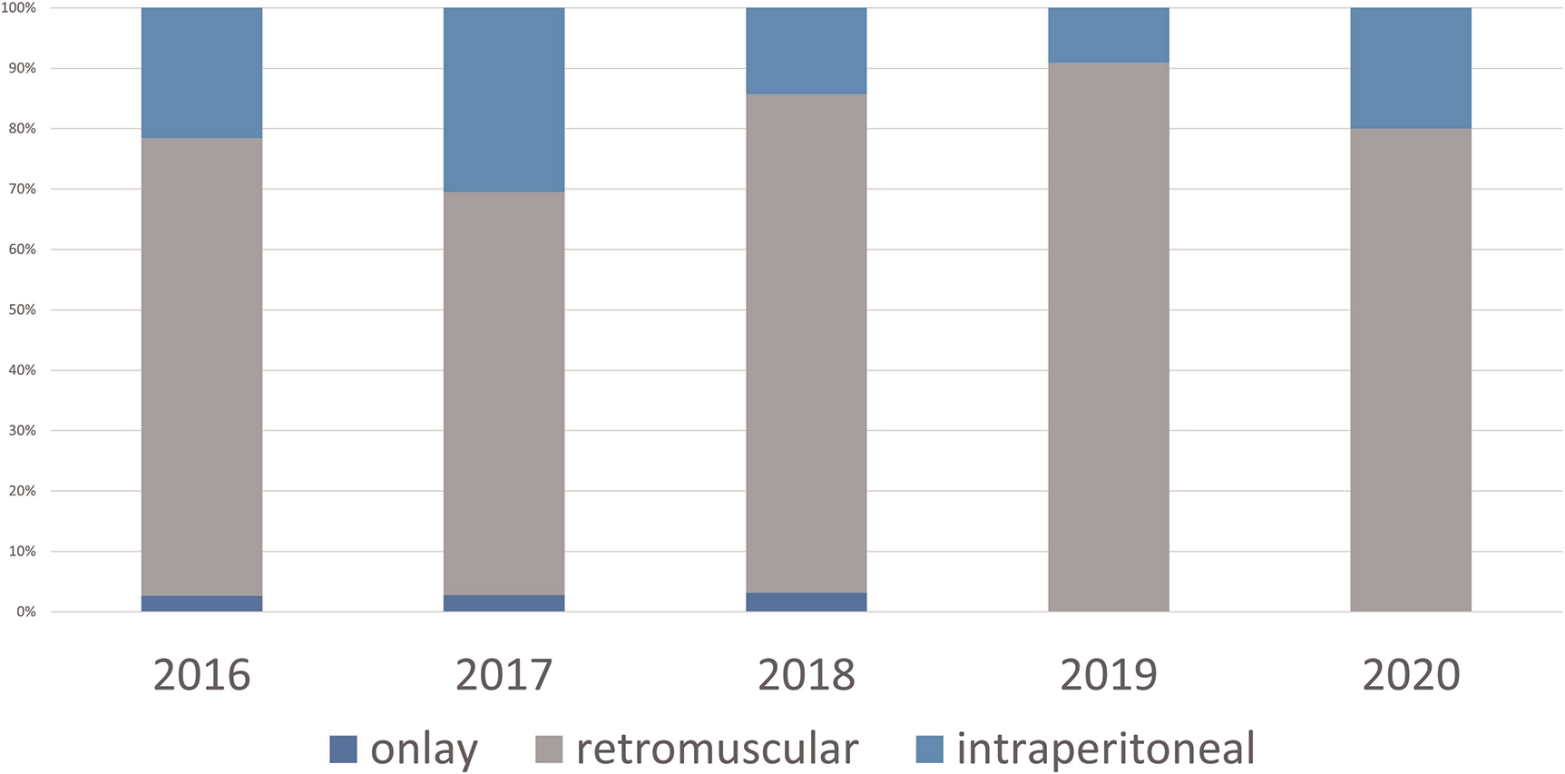
Mesh position open surgery.

**Figure 3:**
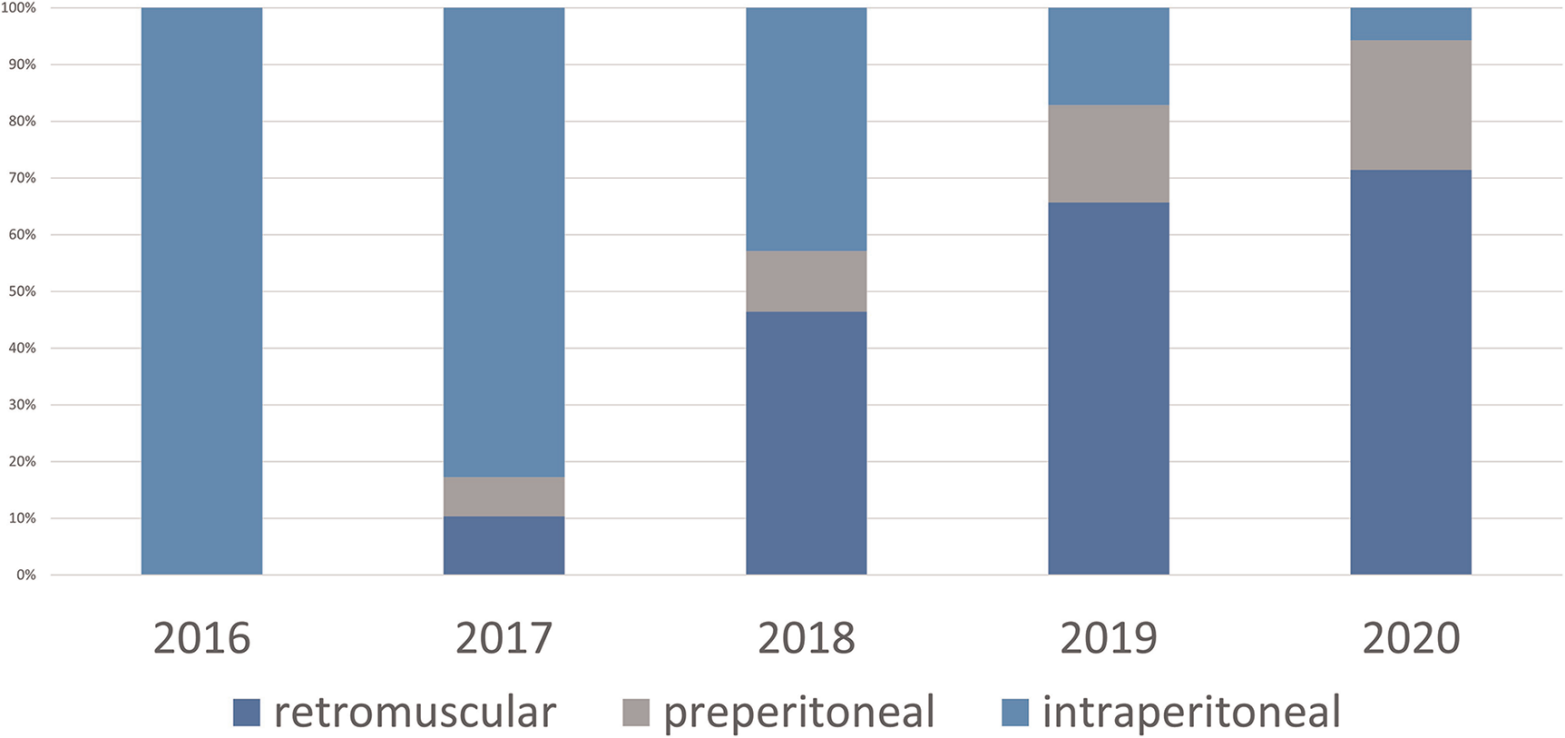
Mesh position minimally invasive surgery.

From January 2016 to December 2020, 312 patients underwent surgery for ventral hernia repair who met the inclusion criteria. The annual interventions steadily increased from 59 in 2016 to 91 in 2018, but then dropped again starting in 2019 and were only 40 in 2020 (Table 1). In 2016 37 of the 59 patients (62.7%) underwent open surgery (Figure 1), in which retromuscular mesh was implanted in 75.7%, intraperitoneal mesh in 21.6%, and onlay mesh in 2.7% (Figure 2). The proportion of patients operated on laparoscopically was 37.3%, with an intraperitoneal mesh implanted in all cases (Figure 3).

**Table 1:** Development of mesh position, complications and complexity of the ventral hernia repair in the period of 2016 to 2020, M, medial defect, L, lateral defect, C, combined medial and lateral defect, W3, width of the defect more than 10 cm.

	2016			2017			2018			2019				2020			
	Open	Laparoendo scopic	Total	Open	Laparoendo scopic	Total	Open	Laparoendo scopic	Total	Open	Laparoendo scopic	Robotic	Total	Open	Laparoendo scopic	Robotic	Total
Onlay	1	0	1	1	0	1	2	0	2	1	0	0	1	0	0	0	0
	2.70%	0.00%	1.69%	2.78%	0.00%	1.54%	3.17%	0.00%	2.20%	5.00%	0.00%	0.00%	1.82%	0.00%	0.00%	0.00%	0.00%
Retromuscular	28	0	28	24	3	27	52	13	65	17	11	12	40	4	0	25	29
	75.68%	0.00%	47.46%	66.67%	10.34%	41.54%	82.54%	46.43%	71.43%	85.00%	64.71%	66.67%	72.73%	80.00%	0.00%	83.33%	72.50%
Preperitoneal	0	0	0	0	2	2	0	3	3	0	1	5	6	0	3	5	8
	0.00%	0.00%	0.00%	0.00%	6.90%	3.08%	0.00%	10.71%	3.30%	0.00%	5.88%	27.78%	10.91%	0.00%	60.00%	16.67%	20.00%
Intraperitoneal	8	22	30	11	24	35	9	12	21	2	5	1	8	1	2	0	3
	21.62%	100.00%	50.85%	30.56%	82.76%	53.85%	14.29%	42.86%	23.08%	10.00%	29.41%	5.56%	14.55%	20.00%	40.00%	0.00%	7.50%
Total	37	22	59	36	29	65	63	28	91	20	17	18	55	5	5	30	40
Overall complications	7	2	9	9	4	13	9	5	14	2	2	2	6	1	1	4	6
	18.92%	9.09%	15.25%	25.00%	13.79%	20.00%	14.29%	17.86%	15.38%	10.00%	11.76%	11.11%	10.91%	20.00%	20.00%	13.33%	15.00%
Intraoperative complications	1	0	1	1	1	2	1	0	1	1	0	0	1	0	0	0	0
Clavien Dindo I	3	1	4	2	2	4	4	4	8	0	2	0	2	1	1	2	4
Clavien Dindo II	1	0	1	1	0	1	1	0	1	0	0	0	0	0	0	0	0
Clavien Dindo IIIa	0	0	0	0	0	0	0	0	0	0	0	1	1	0	0	0	0
Clavien Dindo IIIb	2	1	3	3	1	4	2	0	2	0	0	1	1	0	0	1	1
Clavien Dindo IVa	0	0	0	1	0	1	1	1	2	1	0	0	1	0	0	1	1
Clavien Dindo IVb	0	0	0	0	0	0	0	0	0	0	0	0	0	0	0	0	0
Clavien Dindo V	0	0	0	1	0	1	0	0	0	0	0	0	0	0	0	0	0
Reoperations	2	1	3	3	1	4	2	0	2	0	0	1	1	0	0	1	1
	5.41%	4.55%	5.08%	8.33%	3.45%	6.15%	3.17%	0.00%	2.20%	0.00%	0.00%	5.56%	1.82%	0.00%	0.00%	3.33%	2.50%
Complexity	10	2	12	10	4	14	12	10	22	2	8	5	15	1	3	10	14
	27.03%	9.09%	20.34%	27.78%	13.79%	21.54%	19.05%	35.71%	24.18%	10.00%	47.06%	27.78%	27.27%	20.00%	60.00%	33.33%	35.00%
EHS M	31	22	53	30	26	56	61	22	83	17	15	12	44	4	2	23	29
	83.78%	100.00%	89.83%	83.33%	89.66%	86.15%	96.83%	78.57%	91.21%	77.27%	88.24%	66.67%	77.19%	80.00%	40.00%	76.67%	72.50%
EHS L	4	0	4	5	3	8	2	6	8	2	2	5	9	1	3	4	8
	10.81%	0.00%	6.78%	13.89%	10.34%	12.31%	3.17%	21.43%	8.79%	9.09%	11.76%	27.78%	1579.00%	20.00%	60.00%	13.33%	20.00%
EHS ML (combined)	2	0	2	1	0	1	0	0	0	1	0	1	2	0	0	3	3
	5.41%	0.00%	3.39%	2.78%	0.00%	1.54%	0.00%	0.00%	0.00%	4.55%	0.00%	5.56%	3.51%	0.00%	0.00%	10.00%	7.50%
EHS W3	2	0	2	7	0	7	6	2	8	4	0	1	5	0	0	6	6
	5.41%	0.00%	3.39%	19.44%	0.00%	10.77%	9.52%	7.14%	8.79%	18.18%	0.00%	5.56%	8.77%	0.00%	0.00%	20.00%	15.00%

The proportion of patients which had been operated on using a minimally invasively approach initially increased steadily to 44.6% in 2017, the year in which minimally invasive procedures with extraperitoneal mesh placement were started. In the following year, extraperitoneal mesh placement was performed in 74.7% of all patients (68 of 91 patients) and thus more than half of all minimally invasive patients (46.4% retromuscular mesh placement, 10.7% preperitoneal mesh placement), with the proportion of minimally invasive patients decreasing again to 30.77% in 2018 due to the “comeback” of open sublay technique.

**Figure 4:**
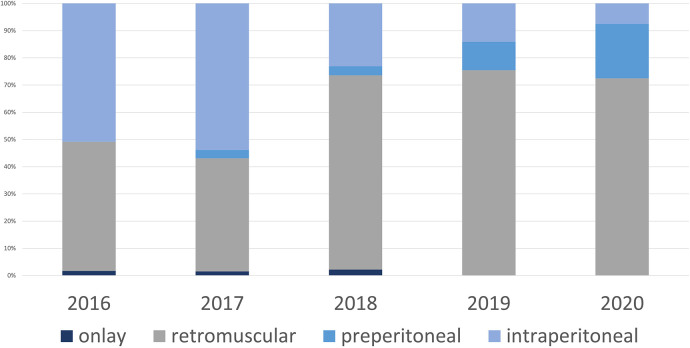
Mesh position all procedures.

With the availability of the robot starting in 2019, the percentage of minimally invasive patients (laparoendoscopic and robotic) operated on increased further to 87.5% in 2020. In 94.3% of minimally invasive patients (33 of 35 patients) extraperitoneal mesh placement could be performed (71.4% retromuscular mesh placement, 25 of 35 patients, 22.86% preperitoneal mesh placement, 8 of 35 patients). Overall, the percentage of extraperitoneal meshes placed in the preperitoneal and retromuscular regions increased from 47.5% to 92.5% during the observation period (Figure 4).

**Figure 5:**
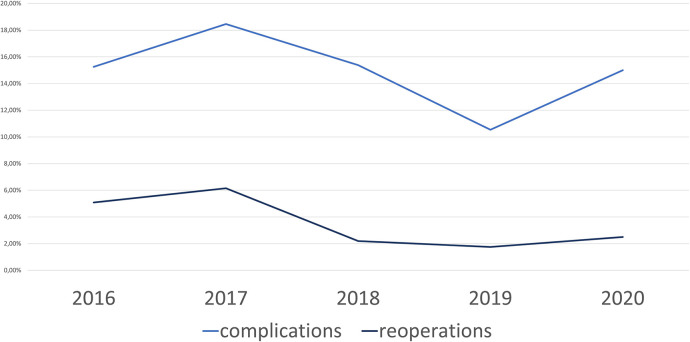
Complications and reoperations all procedures.

The complication rate of all operative techniques fluctuated around 15% (Figure 5). The robotic group had a complication rate slightly lower with 11.1% in 2019 and 13.3% in 2020. Details of the complications according to the type of surgery can be found in Table 2. The reoperation rate within the first 6 weeks after surgery decreased over the observation period from 5.1 to 2.5%. The percentage of complex cases increased over time from 20.3% in 2016 to 35.0% in 2020. In 2020, 5 patients were operated on using the open technique. In 4 of these cases an emergency situation was present. In the only elective case that underwent open surgery, a simultaneous abdominoplasty was performed.

**Table 2:** List of complications according to the type of surgery, o-TAR, open transversus abdominis release, o-RS, open rives-stoppa, o-IPOM, open intraperitoneal onlay mesh, o-onlay, open onlay mesh, l-IPOM, laparoendoscopic intraperitoneal onlay mesh, l-eTEP, laparoendoscopic enhanced-view totally extaperitoneal plasty, l-eMILOS, laparoendoscopic enhanced-view mini or less open sublay, l-ventralTAPP, laparoendoscopic ventral transabdominal preperitoneal plasty, r-ventralTAPP, robotic ventral transabdominal preperitoneal plasty, r-eTEP, robotic enhanced-view totally extaperitoneal plasty, r-TARUP, robotic transabdominal retromuscular umbilical prosthetic hernia repair, r-IPOM, robotic intraperitoneal onlay mesh, r-TAR, robotic transversus abdominis release, in parentheses after the complications the degree of severity according to the clavien dindo classification.

	2016		2017		2018		2019		2020	
Type of surgery	Number of prcedures	Type of complication	Number	Type of complication	Number	Type of complication	Number	Type of complication	Number	Type of complication
o-TAR	0		3	Seroma (III b), haematoma (III b) and death (V)	4		4	Pulmonary embolism (IV a)	0	
o-RS	28	Renal failure (I), haematoma (I), pneumonia (II), intraoperative bowel injury	21	Haematoma (III b), minor wound problem (I), ileus (I), non-ST-elevation-infarction (IV a), pneumonia (II)	48	Mesh infection (III b), wound infection (III b), seroma (I), haematoma (I), pulmonary embolism (IV a), minor wound problem (I), intraoperative bowel injury	13	Intraoperative bowel injury	4	Seroma (I)
o-IPOM	8	Mesh infection (III b), minor wound problem (I)	11		9	pneumonia (II), haematoma (I)	2		1	
o-onlay	1	Wound infection (III b),	1	Intraoperative bowel injury	2		1		0	
l-IPOM	22	Bowel obstruction (III b), gastroenteritis (I), intraoperative bowel injury	24	Haematoma (III b), haematoma (I), seroma (I)	12		5	Seroma (I)	2	
l-eTEP	0		2		11	Paresis of the radial nerve (I), 3 x seroma (I), pulmonary embolism (IV a)	8	Seroma (I)	0	
l-eMILOS	0		1		2		3		0	
l-ventral TAPP	0		2		3		1		3	Haematoma (I)
r- ventral TAPP	0		0		0		5	Haematoma (III b)	5	Ileus (III b)
r- eTEP	0		0		0		6	Bleeding (III a)	17	Pankreatitis (I), minor wound problem (I)
r-TARUP	0		0		0		6		2	
r-IPOM	0		0		0		1		0	
r-TAR	0		0		0		0		6	Pulmonary embolism (IV a)
total	59		65		91		55		40	

## Discussion

About 50,000 incisional hernias have been operated on annually in Germany consistently over the last few years (19). Every clinic is confronted with the therapy of this frequent clinical diagnosis. The treatment of abdominal wall hernias has undergone some significant changes in the last decade, concerning mesh position on one hand and surgical approach on the other.

In contrast to other areas of abdominal surgery, Köckerling reported a decreasing trend of minimally invasive surgery in favor of the open sublay procedure in his recent analysis of the Herniamed database (4).

These changes can also be seen in our data. While the percentage of patients who received laparoscopic IPOM surgery increased in the first years of the observation period before laparoscopic techniques with extraperitoneal mesh placement were started to 36.9% (24 of 65 patients) in 2017, the number decreased continuously from this date to only 5% (2 of 40 patients) by 2020. The decrease in laparoscopic IPOM procedures temporarily led to an increase in open retromuscular procedures, which increased to 57.14% (52 of 91 patients) in 2018.

This changeover is attributed to the mesh placement, as minimally invasive ventral hernia repair has been previously associated with intraperitoneal mesh positioning. Therefore instead of comparing the robotical and the laparascopical approach, as it is conveniently done in colorectal surgery, the robotic operation should be rather compared to the open approach applying retromuscular mesh placement.

In 2020, Lu et al. published the only study comparing a laparoscopic with a robotic extraperitoneal surgical procedure (eTEP) with retromuscular mesh position (20). The outcome of the two surgical procedures was comparable. But because the patient groups differed significantly and the robotic group included more complex hernia findings as well as patients with higher BMI and ASA status, the authors concluded that the use of the robot would expand the spectrum of minimally invasive hernia repair.

In 2021, the first meta-analysis of robotic hernia procedures with retromuscular mesh position was published (21). Santos described 4 evidence-based principles of hernia surgery: mesh reinforcement, retromuscular mesh position without mesh fixation, primary fascial closure and minimally invasive technique. These conditions were met in their entirety only by using robotic hernia procedures. The use of the robot significantly eases the previous difficulties of suture closure in confined spaces.

Minimally invasive ventral hernia repair with retromuscular mesh placement was started in our hospital in 2017. In the initial stages laparoscopic surgeries were performed mainly on small to medium-sized findings with an EHS width of 1 to 2.

With the availability of robotics, the percentage of minimally invasive procedures with extraperitoneal mesh placement increased to 82.5% in 2020, now including more complex findings, such as EHS W3 hernias.

In 2021 Muysoms showed very similar results in his ROBUST hernia project (22). In 451 patients undergoing incisional hernia repair the portion of laparoscopic IPOM surgery decreased from 52% in 2015 to 14% in 2019. In the same time the robotic access which was performed since 2016 increased to 75% in 2019. The authors are confident that the main clinical value of the robotic approach in ventral hernia repair is the treatment of complex hernias, as for example in wide incisional hernias which require a component separation.

To this date, there is still no randomized controlled trial (RCT) comparing robotic- to open hernia surgery with retromuscular mesh placement. In 2021 a metaanalysis on this topic by Bracale et al. analysing 237 robotic vs. 594 open TARs showed a significantly reduced overall complication rate in the robotic group (9.3%) compared to the open group (20.7%) as well as a trend to a lower surgical side infection (SSI) rate in the robotic group (3.6% rTAR vs. 5.2% oTAR) (23). The authors are convinced that robotic TAR improves recovery by adding the benefits of minimally invasive procedures when compared to open surgery.

Currently, it cannot be concluded from these data that robotic surgery is superior to open surgery. It remains to be seen whether future RCTs will show a difference. Since the advantages of minimally invasive surgery have already been demonstrated in other areas of surgery (24), it can be expected that they will also be demonstrated in this area, which has not yet been accessible to minimally invasive care.

Our results show a decreasing reoperation rate (2.5% in 2020) and a stable complication rate between 10% and 18% over the last 5 years including all kinds of ventral hernias containing a considerable number of complex hernias, which steadily increased to 35% in 2020. It could be shown that the extension of the surgical spectrum using robotics is not associated with an increased complication or reoperation rate.

Referring to literature the complication rate of robotic hernia surgery is similar to our results. In an evaluation of the AHSQC database on real world evidence by La Pinska et al., a comparison between robotic and laparoscopic hernia surgery excluding patients with parastomal hernia and TAR, which was not excluded from our study, showed a postoperative complication rate of 10% vs. 11% (25).

Limitations of this study are its retrospective design and the grouping of different surgical techniques under one umbrella term of surgical access. Our paper is a purely descriptive analysis of the conversion process, without comparisons to own or other data. When robotics was started in 2019 the number of patients treated for ventral hernias decreased as the operation time ventral hernia repair using a robotically-assisted approach is much longer especially during the learning curve. Hence less patients could be treated in the same amount of time and the overall operating capacity could not be increased due to limited personnel resources. In the year 2020 the number of robotic interventions decreased further due to the COIVD19-pandemic and the resulting shortages in resources and personnel.

Based on the abdominal wall procedures performed in the 5 consecutive years from 2016 to 2020, we demonstrate that the majority of ventral hernia procedures can be performed safely in a minimally invasive technique with extraperitoneal mesh placement using the robot without leading to an increase in complications. Robotically assisted hernia repair is a promising new technique that is also practical for complex hernias.

## Data Availability

The raw data supporting the conclusions of this article will be made available by the authors, without undue reservation.
